# *Ganoderma lucidum* polysaccharides attenuates pressure-overload-induced pathological cardiac hypertrophy

**DOI:** 10.3389/fphar.2023.1127123

**Published:** 2023-03-23

**Authors:** Changlin Zhen, Xunxun Wu, Jing Zhang, Dan Liu, Guoli Li, Yongbo Yan, Xiuzhen He, Jiawei Miao, Hongxia Song, Yifan Yan, Yonghui Zhang

**Affiliations:** ^1^ Chongqing Key Laboratory of Development and Utilization of Genuine Medicinal Materials in Three Gorges Reservoir Area, Chongqing, China; ^2^ Chongqing Engineering Research Center of Antitumor Natural Drugs, Chongqing Three Gorges Medical College, Chongqing, China; ^3^ School of Biomedical Science, Huaqiao University, Quanzhou, China; ^4^ The People’s Hospital Affiliated to Chongqing Three Gorges Medical College, Chongqing, China

**Keywords:** GLPs, heart function, PPARγ, PGC-1α, cardiac hypertrophy

## Abstract

Pathological cardiac hypertrophy is an important risk factor for cardiovascular disease. However, drug therapies that can reverse the maladaptive process and restore heart function are limited. *Ganoderma lucidum* polysaccharides (GLPs) are one of the main active components of *G. lucidum* (*Ganoderma lucidum*), and they have various pharmacological effects. GLPs have been used as Chinese medicine prescriptions for clinical treatment. In this study, cardiac hypertrophy was induced by transverse aortic constriction (TAC) in mice. We found that GLPs ameliorate Ang II-induced cardiomyocyte hypertrophy *in vitro* and attenuate pressure overload–induced cardiac hypertrophy *in vivo*. Further research indicated that GLPs attenuated the mRNA levels of hypertrophic and fibrotic markers to inhibit cardiac hypertrophy through the PPARγ/PGC-1α pathway. Overall, these results indicate that GLPs inhibit cardiac hypertrophy through downregulating key genes for hypertrophy and fibrosis and attenuate pressure overload-induced pathological cardiac hypertrophy by activating PPARγ. This study provides important theoretical support for the potential of using GLPs to treat pathological myocardial hypertrophy and heart failure.

## 1 Introduction

Cardiovascular disease is one of the major threats to human life and health, and cardiac hypertro-phy is an important contributor (Braunwald, 2015). Hypertension, aortic stenosis, and other conditions causing long-term increases in ventricular afterload are common risk factors for cardiac hypertrophy (Oldfield et al., 2020). Among the many ways to treat and prevent cardiac hypertrophy, medication is effective because of good patient compliance and low patient costs. Although current drug therapies are effective in relieving symptoms, their effects on disease prevention and reducing morbidity and mortality are limited. Therefore, finding new drugs by studying homologous medicine and food in detail could be key to inhibiting cardiac hypertrophy.

Cardiac hypertrophy is an adaptive response to mechanical stimuli, such as pregnancy, exercise training, and pressure overload, and it can be classified as pathological or physiological cardiac hypertrophy according to the different causes (Tham et al., 2015; Nakamura and Sadoshima, 2018). Physiological cardiac hypertrophy, which is induced by pregnancy and athletic training, is reversible and does not lead to heart failure (Gibb et al., 2017). However, pathological cardiac hypertrophy, which is characterized by heart fibrosis and failure, is irreversible (Yang et al., 2020). In the pathogenesis of pathological cardiac hypertrophy, the cardiomyocyte area increases due to long-term high workload, leading to ventricular wall thickening; persistent pressure overload can lead to the heart entering a decompensated state with cardiomyocyte apoptosis and fibrosis, eventually leading to heart failure (Yang et al., 2021). Although many pathogenesis and molecular mechanisms of cardiac hypertrophy have been described, therapeutic strategies for preventing and managing this pathological process remain limited. Peroxisome proliferator-activated receptor gamma (PPARγ) and its coactivator 1α (PGC-1α) play critical roles in controlling cardiac metabolism (Marian and Braunwald, 2017; Li et al., 2018). PPARγ activation improves cardiac hypertrophy and cardiometabolic dysfunction (Wang et al., 2012; Ni et al., 2020). Relevant studies in rats fed a high-fat diet have confirmed that metformin can attenuate cardiac hypertrophy *via* the PPARγ signaling pathway (Liu et al., 2022). Other literature has reported that PPAR𝛾 signaling inhibits cardiac hypertrophy by activating autophagy (Yuan et al., 2017). Additionally, many traditional Chinese medicines have been shown to attenuate cardiac hypertrophy by upregulating PPAR𝛾 (Gao et al., 2018; Ni et al., 2020; Zhou et al., 2021). Moreover, activating PPARγ improved myocardial cell injury by reducing myocardial fibrosis and cardiomyocyte apoptosis induced by ischemia/reperfusion (Ma et al., 2017; Peng et al., 2017; Zhou et al., 2017). Therefore, the identification of potential PPARγ activators has great potential research value for the treatment of cardiac hypertrophy.

*Ganoderma lucidum* (*Ganoderma lucidum*) is a mushroom belonging to the Polyporaceae family of Basidiomycota, and it has been used as a traditional medicine for more than 2000 years, particularly in Asian countries; the fruiting bodies, culture mycelia, and spores of *G. lucidum* contain various bioactive chemicals, such as polysaccharides, triterpenes, and proteins (Gong et al., 2020). *Ganoderma lucidum* polysaccharides (GLPs) are one of the main active components, and they have various pharmacological effects. In fact, GLPs have protective anti-free radical effects and can reduce cell damage caused by mutagens, in addition to their immune enhancing and anti-tumor effects (Jiang et al., 2017; Huang et al., 2019; Zhang et al., 2021). Some studies have shown that GLPs can protect the cardiovascular system by preventing cardiomyocyte damage. GLPs protect the myocardium by reducing MDA, activating antioxidant enzymes (GSH-Px, CAT, SOD and NO) in heart tissue, and reducing lipid peroxidation in type 2 diabetic rats (Xue et al., 2010). Patients with coronary heart disease (CHD) treated with GLPs for 12 weeks had improved CHD symptoms and decreased mean blood pressure values (Yihuai et al., 2004). In patients with atrial fibrillation, GLPs were shown to have a cardioprotective effect, manifested by a significant decrease in systolic and diastolic blood pressure, heart rate, and inflammatory factors such as LDL-C, IL-1b, IL-6, hsCRP, and TNF-a (Rizal et al., 2020). However, little is known of the function of GLPs in cardiac hypertrophy.

Our study aims to determine whether GLPs treatment can ameliorate cardiac hypertrophy by using histopathological and molecular biology methods *in vivo* and *in vitro*. Furthermore, we will investigate the mechanism of GLPs regulating cardiac hypertrophy.

## 2 Materials and methods

### 2.1 Transverse aortic constriction

Transverse aortic constriction (TAC) was implemented as described previously (Jiang et al., 2014). In narcotized mice, the aorta was ligated with a ligation line that was tied tightly. The constrictive band was placed around the aortic arch between the innominate and the left common carotid arteries. In sham mice, the ligation was not performed.

### 2.2 Animals

Eight-week-old C57BL/6J male mice were obtained from CavensBiogle (Changzhou, China) and maintained in specific pathogen-free rooms at a constant temperature (20°C–26°C) and humidity (40%–70%). Additionally, they were maintained on a 12-h light/dark cycle and had free access to food (normal chow) and water. Mice were randomly assigned to each experimental group. GLPs were procured from ESITEBiogle (Chengdu, China; purity: UV ≥ 95%) and dissolved in sterile water. TAC was used to induce cardiac hypertrophy in the mice. In brief, in narcotized mice, a constrictive band was placed around the aortic arch between the innominate and left common carotid arteries. We determined the GLP and PPARγ inhibitor doses *in vivo* and *in vitro* by consulting the literature and performing preliminary experiments (Li et al., 2011; Ni et al., 2020; Hu et al., 2022). GLPs at a dose of 100 mg/kg/d were administered to the mice for 4 weeks *via* oral gavage. Simultaneously, the mice received intraperitoneal injections of the PPARγ inhibitor T0070907 at a dose of 1 mg/kg/d. The mice were placed randomly into the following experimental groups, with four animals in each group: Sham, TAC, Sham + GLPs, TAC + GLPs, and Sham + T0070907 + GLPs + TAC group. All animal experiments were performed with approval from the Institutional Animal Care and Use Committee of the Chongqing Three Gorges Medical College (no: A2022015).

### 2.3 Cell culture and treatments

H9C2 cells were cultured with 10% fetal bovine serum (Solarbio, Beijing, China), high-glucose-DMEM (Solarbio, Beijing, China) and antibiotics. GLPs were procured from SEITEbiogle (Chengdu, China). Angiotensin II was from Sigma (A9525, St. Louis, MO, United States). Antibodies against the following proteins were used: PPARγ (Abcam, ab272718, London, United Kingdom, 1:1000), PGC1α (Abcam, ab176328, London, United Kingdom, 1:1000), and β-actin (NCM Biotech, AB1020, Suzhou, Jiangsu, China, 1:1000). BCA protein assay kit was purchased from Beyotime (Shanghai, China), and T0070907 was obtained from Selleck (Houston, Texas, United States).

### 2.4 Echocardiography

The mice were anesthetized and imaged using a Vevo 3100 Ultrasound (Visual Sonics, Toronto, Ontario, Canada) after TAC or Sham surgery. The instrument instructions were followed to detect the end-diastolic diameter (LVID; d), left ventricular end-systolic diameter (LVID; s), left ventricular ejection fraction (EF%), and fractional shortening (FS%) of the mouse hearts.

### 2.5 Histology

We flushed the mouse hearts with PBS and fixed them with 4% paraformaldehyde. As described in a previous study, the cross-sectional surface area of cardiomyocytes was measured by hematoxylin and eosin (H&E) staining (Hasan et al., 2018). Fifty cardiomyocytes were counted per heart, and the average area was calculated. Heart fibrosis was detected with Masson’s trichrome (He et al., 2017).

### 2.6 Immunofluorescence microscopy

Immunofluorescence staining was performed as described previously (He et al., 2015b). In brief, after treatment with the indicated drugs, the cardiomyocytes were incubated with ɑ-actinin (Proteintech, 11313-2-AP, Wuhan, Hubei, China, 1:500), followed by AffiniPure donkey anti-rabbit IgG (H + L) fluorescent secondary antibody (Jackson ImmunoResearch, 1:200). A fluorescence microscope was used to observe the cell area, and Image-Pro Plus software was used to quantify the cardiomyocyte area.

### 2.7 Western blotting

The cells and mouse heart tissue were homogenized in lysis buffer to extract whole-cell lysates, and the protein concentrations of the samples were determined by BCA kit. The cleavage products were examined by Western blotting assay as described previously (He et al., 2015a). The protein concentration of each sample was adjusted to the same level, and 10 μL of each sample was loaded into a gel for electrophoresis. The protein samples were then separated by SDS-PAGE, and the proteins in the gel were transferred electrically to a polyvinylidene fluoride (PVDF) membrane. Non-specific binding was blocked by incubating the membranes in 5% BSA. Afterwards, the PVDF membranes and antibodies were incubated at 4°C overnight, and the corresponding horseradish peroxidase secondary antibody (ZSGB-BIO, goat anti-rabbit ZB-2301) was added for 1 h. The membranes were washed, and the images were recorded and analyzed with a Bio-Rad Bole ChemiDoc MP Chemiluminescent Gel Imaging System. Antibodies against the following proteins were used: PPARγ (Abcam, ab272718, London, United Kingdom, 1:1000), PGC1α (Abcam, ab176328, London, United Kingdom, 1:1000), and β-actin (NCM Biotech, AB1020, Suzhou, Jiangsu, China, 1:1000).

### 2.8 Quantitative real time-PCR

Total RNA was extracted from mouse myocardial tissue and cardiomyocytes using Trizol (Beyotime, Shanghai, China) according to the manufacturer’s instructions. cDNA was synthesized with a cDNA first-strand synthesis kit (Yeasen Biotech, Shanghai, China). An ABI 9500 Real-Time PCR system (Applied Biosystems, Carlsbad, United States) and Hieff UNICON® universal Blue qPCR SYBR Green Master Mix (Yeasen, Shanghai, China) were used to perform quantitative real-time PCR. Gapdh was used as an internal control. The relative mRNA expression was calculated with the 2^-△△Ct^ method. The primers used in the present study are listed in Table 1.

**TABLE 1:** Sequences of primers used in quantitative real-time polymerase chain reaction.

Gene name	Forward primer	Reverse primer
Anp (rat)	5'-GAG​AAG​ATG​CCG​GTA​GAA​GAT​G-3'	R:5'-ACTTAGCTCCCTCTCTGAGG-3'
Bnp(rat)	5'-CTG​CTG​GAG​CTG​ATA​AGA​GAA​A-3'	5'-GCG​CTG​TCT​TGA​GAC​CTA​A-3'
β-MHC(rat)	F:5'-CCAACACCAACCTATCCAA-3'	R:5'-GCCAATGTCACGGCTCTT-3'
Gapdh (rat)	F:5’-CATCTCCCTCACAATTCCATCC-3'	R:5'-GAGGGTGCAGCGAACTTTAT-3'
Anp (mouse)	F:5'-TCCGATAGATCTGCCCTCTT-3'	R:5'-CTCCAATCCTGTCAATCCTACC-3'
Bnp(mouse)	F:5'-ACCACCTTTGAAGTGATCCTATT-3'	R:5'-GCAAGTTTGTGCTCCAAGATAAG-3'
β-MHC(mouse)	F:5'-CCGAGTCCCAGGTCAACAA-3'	R:5'-CTTCACGGGCACCCTTGGA-3'
Gapdh (mouse)	F:5'-GTGGCAAAGTGGAGATTGTTG-3'	R:5'-CGTTGAATTTGCCGTGAGTG-3'
α-SMA (mouse)	F:5'-AGGGAGTGATGGTTGGAATG-3'	R:5'-GGTGATGATGCCGTGTTCTA-3'
Col1a1 (mouse)	F:5'-AGGCTTCAGTGGTTTGGATG-3'	R:5'-CACCAACAGCACCATCGTTA-3'
Col3a1 (mouse)	F:5'-CCCAACCCAGAGATCCCATT-3'	R:5'-GAAGCACAGGAGCAGGTGTAGA-3'
Fibronectin (mouse)	F:5'-CCGGTGGCTGTCAGTCAGA-3'	R:5'-CCGTTCCCACTGCTGATTTATC-3'

Anp, Atrial natriuretic peptide; BNP, b-type natriuretic peptide; β-MHC, β-myosin heavy chain; Col1a1, collagen I; Col3a1, collagen III; α-SMA, α-smooth muscle actin.

### 2.9 Statistical analyses

The data are presented as the mean ± SEM. Time course data were analyzed by repeated measures ANOVA, and significant differences were analyzed by one-way ANOVA followed by Bonferroni post-processing analysis. Comparisons between two groups were assessed using Student’s t-test. A value of *p* < 0.05 was considered statistically significant.

## 3 Results

### 3.1 GLPs alleviated angiotensin II-induced cardiomyocyte hypertrophy

**FIGURE 1:**
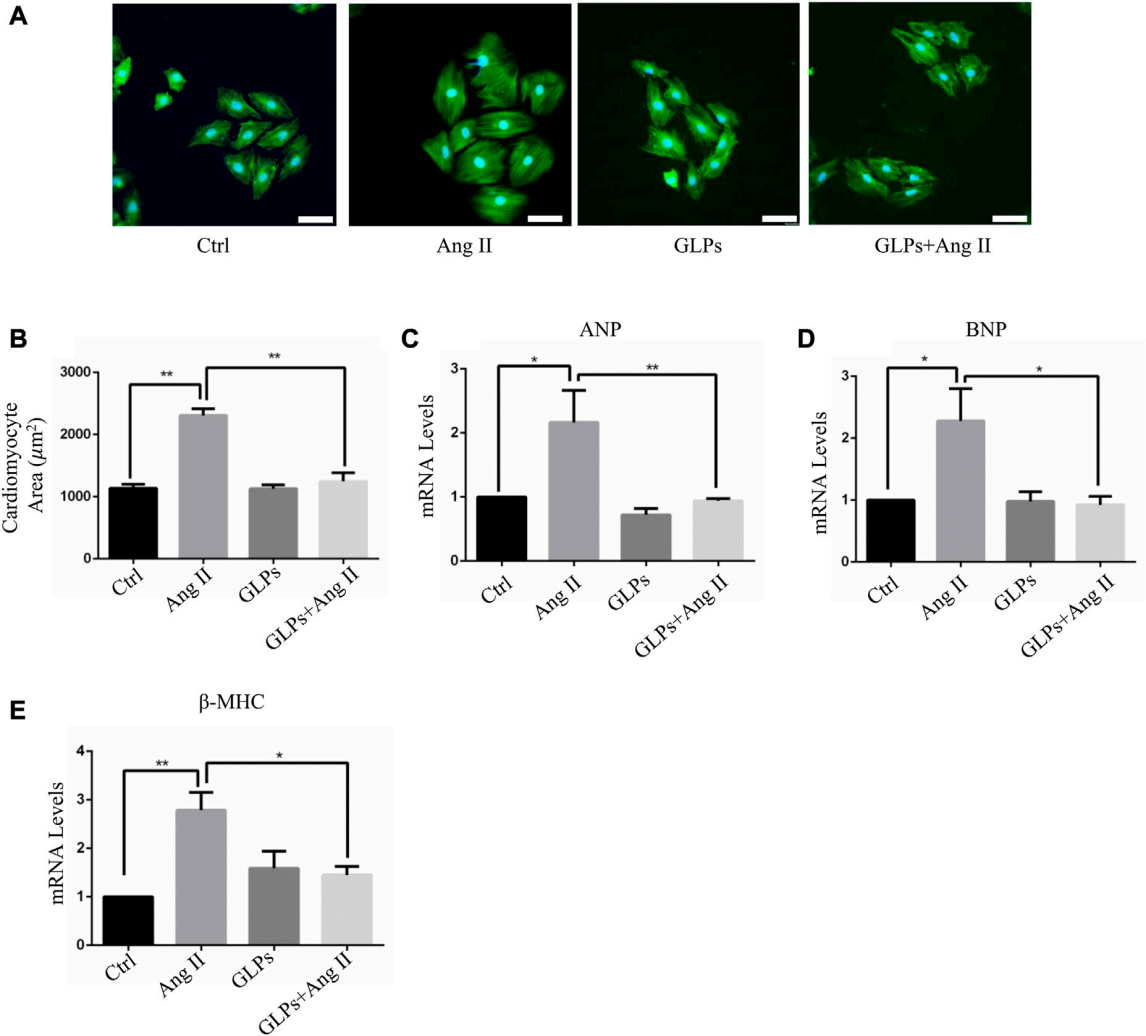
GLPs alleviate Ang II-induced hypertrophy in cardiomyocytes. **(A)** Representative images of cardiomyocytes treated with Ang II (1 µM) and GLPs (50 mg/mL) (green: α-actinin; blue: DAPI). Original magnification ×200, scale bars, 50 μM. **(B)** Quantification of cell surface areas (*n* = 50 + cells in each group). **(C)** Real-time PCR analysis of ANP gene expression in H9C2 cells treated with Ang II and GLPs (*n* = 3). **(D)** Real-time PCR analysis of BNP gene expression in H9C2 cells treated with Ang II and GLPs (*n* = 3). **(E)** Real-time PCR analysis of β-MHC gene expression in H9C2 cells treated with Ang II and GLPs (*n* = 3). All values represent the means ± SEM, *p < 0.05, **p < 0.01. Ctrl: control; Ang II: angiotensin II; ANP: atrial natriuretic peptide; BNP: B-type natriuretic peptide; β-MHC: β-myosin heavy chain; GLPs: *Ganoderma lucidum* polysaccharides.

We first assessed the effect of GLPs on cardiomyocyte hypertrophy induced by angiotensin II (Ang II) in H9C2 cells. The cardiomyocyte area increased after Ang II treatment for 48 h (Figures 1A,B). However, compared with Ang II stimulation, GLPs decreased the cell size (Figures 1A,B). ANP, BNP, and β -MHC are widely considered molecular and biochemical markers of cardiac hypertrophy. Atrial natriuretic peptide (ANP) can reduce cardiac load by decreasing the amount of blood returned to the heart and maintaining cardiac function (Vesely et al., 1994). B-type natriuretic peptide (BNP) has effects similar to those of ANP (Mukoyama et al., 1991). Research has reported that cardiac hypertrophy is associated with increased ANP and BNP expression and changes in the β-myosin heavy chain (MHC) (Bernardo et al., 2010). Thus, we detected hypertrophic marker (ANP, BNP, and β-MHC) mRNA levels in H9C2 cells. Ang II treatment increased ANP, BNP, and β-MHC expression, but these changes were decreased in GLP-treated cardiomyocytes (Figures 1C–E). These data indicate that GLPs ameliorate Ang II-induced cardiomyocyte hypertrophy.

### 3.2 GLPs attenuated pressure overload–induced cardiac hypertrophy *in vivo*

**FIGURE 2:**
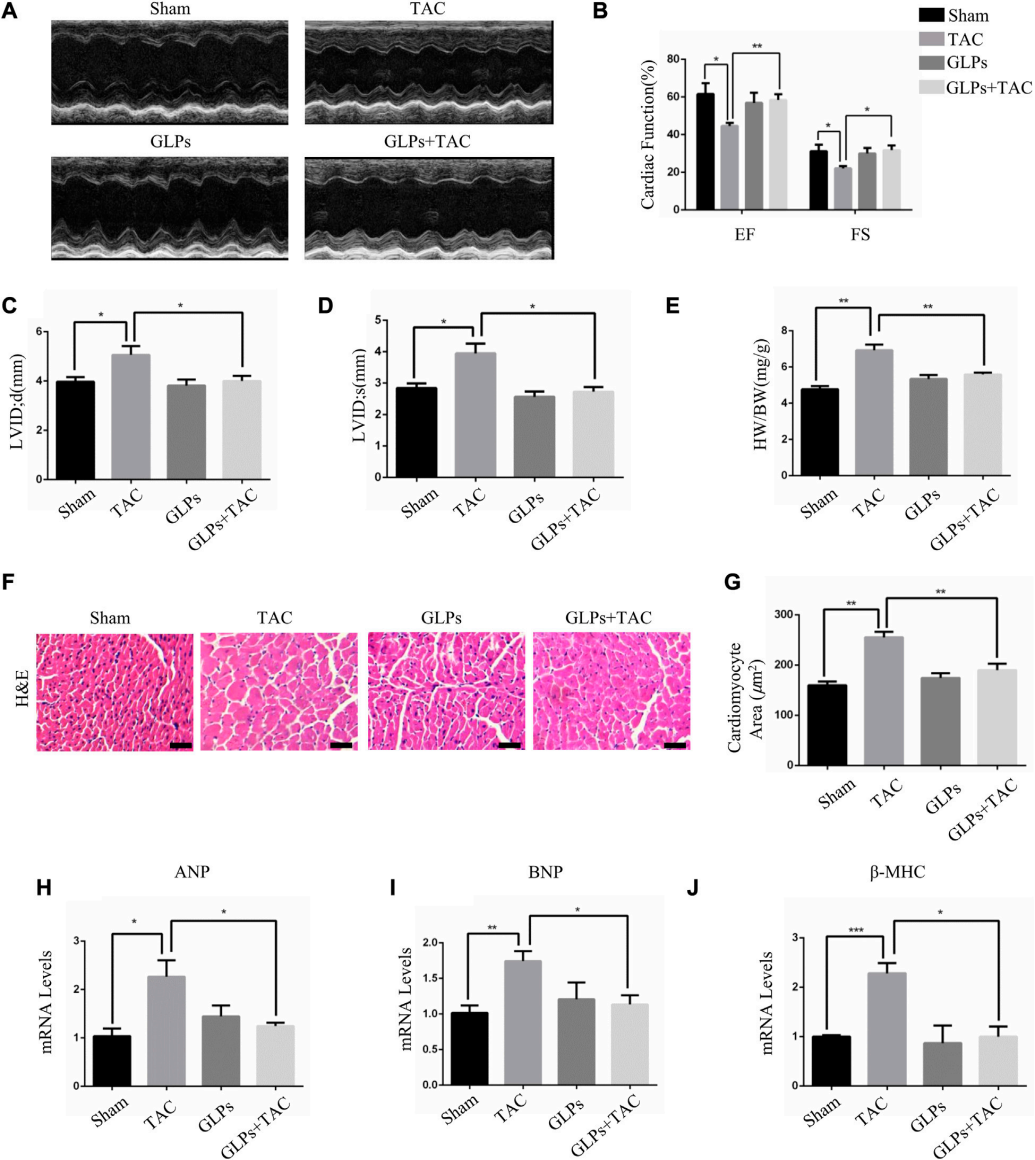
GLPs improve cardiac function and attenuate cardiac hypertrophy induced by TAC *in vivo.* **(A)** Representative M-mode echocardiographic tracings of the left ventricle for mice after sham operation or TAC surgery. **(B)** Quantification of the recovery of EF% and FS% in **(A)** (*n* = 4 per group). **(C)** Quantification of the LVID; d in **(A)**. **(D)** Quantification of the LVID; s in **(A)**. **(E)** Changes in the HW/BW ratio for WT and GLP mice after sham operation or TAC surgery (*n* = 4 per group). **(F)** Histological sections of hearts from WT and GLP mice after sham operation or TAC surgery were stained with H&E to analyze cardiomyocyte size. Original magnification, ×400, scale bars, 100 μM. **(G)** Quantification of cell surface areas (*n* = 50 + cells in each group). **(H–J)** Real-time PCR analysis of hypertrophic markers (ANP, BNP, and β-MHC) in WT and GLP mouse hearts after sham operation or TAC surgery (*n* = 4 per group). All values represent the means ± SEM, *p < 0.05, **p < 0.01. TAC: transverse aortic constriction; EF: ejection fraction; FS: fractional shortening; LVID; d: end-diastolic left ventricular internal dimension; LVID; s: end-systolic left ventricular internal dimension; HW/BW: heart weight/body weight; H&E: hematoxylin and eosin; GLPs: *Ganoderma lucidum* polysaccharides.

**FIGURE 3:**
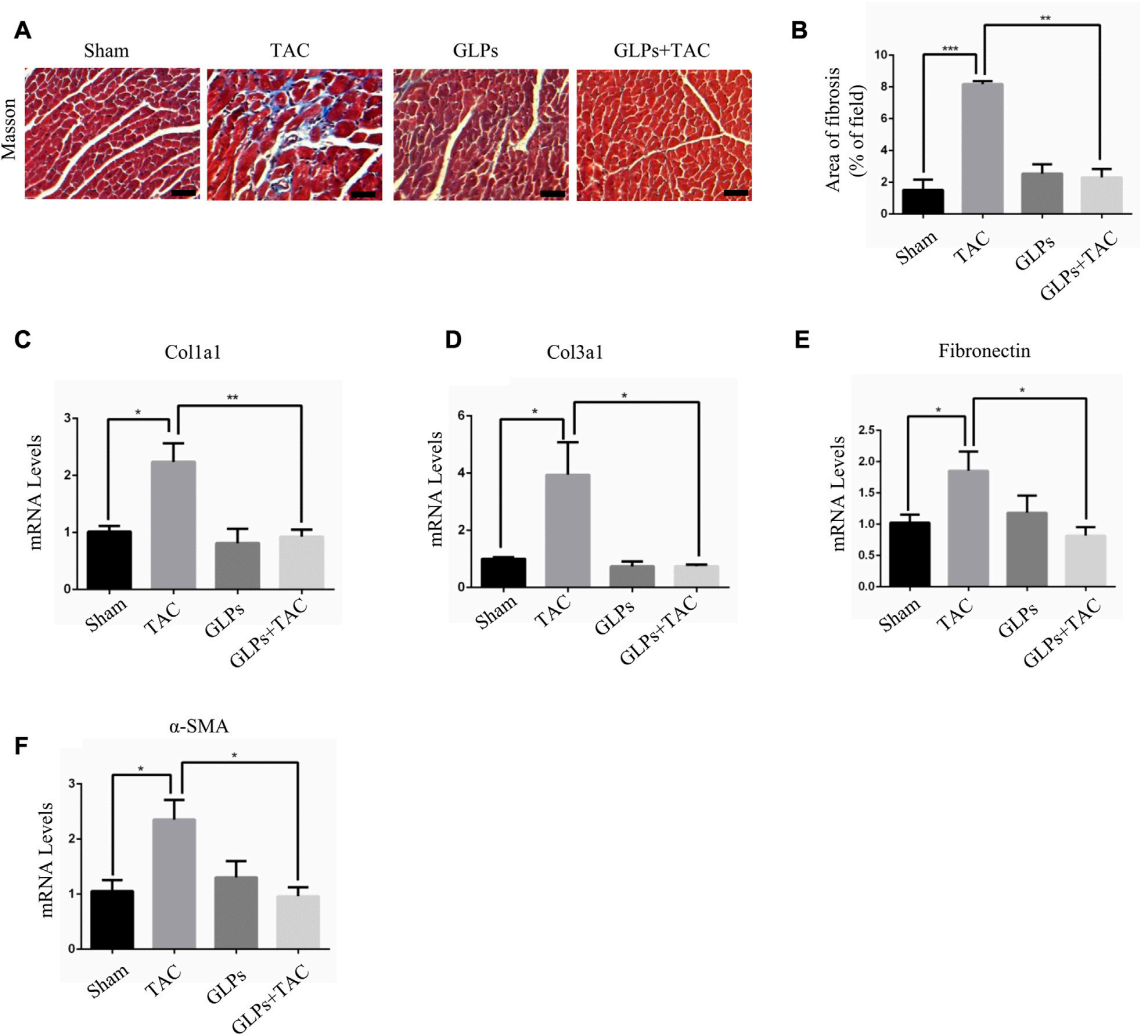
GLPs alleviate cardiac fibrosis induced by TAC *in vivo*. **(A)** Histological sections of hearts from WT and GLP mice after sham operation or TAC surgery were stained with Masson’s trichrome to analyze collagen deposition. Original magnification, ×400, scale bars, 100 μM. **(B)** Statistical results of cardiac interstitial fibrosis (*n* = 4 per group). **(C–F)** Real-time PCR analysis of fibrotic markers (Col1a1, Col3a1, fibronectin, and α-SMA) in WT and GLP mouse hearts after sham operation or TAC surgery (*n* = 4 per group). All values represent the means ± SEM, *p < 0.05, **p < 0.01, ***p < 0.001. WT: wild-type; Col1a1: collagen I; Col3a1: collagen III; α-SMA: α-smooth muscle actin; GLPs: *Ganoderma lucidum* polysaccharides.

*In vivo*, we used transverse aortic constriction (TAC)-induced cardiac hypertrophy to examine the potential role of GLPs in cardiac hypertrophy. After TAC operation, wild-type (WT) mice received an oral gavage of GLPs for 4 weeks. As demonstrated by the echocardiographs shown in Figures 2A–D, WT TAC mouse hearts exhibited a decreased hypertrophic phenotype, evidenced by reduced fractional shortening (FS%) and left ventricular ejection fraction (EF%) and increased end-systolic left ventricular internal dimension (LVID; s) and end-diastolic left ventricular internal dimension (LVID; d). However, GLP treatment prevented deteriorative cardiac hypertrophy as demonstrated by an increase in EF% and FS% and a decrease in LVID; s and LVID; d (Figures 2A–D). The GLP-stimulated group showed attenuated hypertrophic features and decreased heart weight/body weight (HW/BW) ratios (Figure 2E). In the histological analysis, H&E staining showed that the increased cardiomyocyte area induced by TAC was reversed by GLP treatment (Figures 2F,G). Furthermore, GLP treatment decreased the mRNA expression of hypertrophic markers (ANP, BNP, and β-MHC) *in vivo* (Figures 2H–J). Masson’s staining indicated that GLP treatment markedly inhibited cardiac fibrosis relative to that of WT TAC mice (Figures 3A,B). Next, to confirm the antifibrotic effect of GLPs, we measured mRNA levels of fibrotic genes (Col3a1, Col1a1, fibronectin, and α-SMA) by qRT-PCR. GLPs reduced the upregulation of fibrotic genes induced by TAC (Figures 3C–F). Taken together, these results show that GLPs alleviated cardiac hypertrophy, fibrosis, and failure after the TAC operation.

### 3.3 GLPs regulated PPARγ and PGC-1α in cardiomyocytes and mouse hearts

**FIGURE 4:**
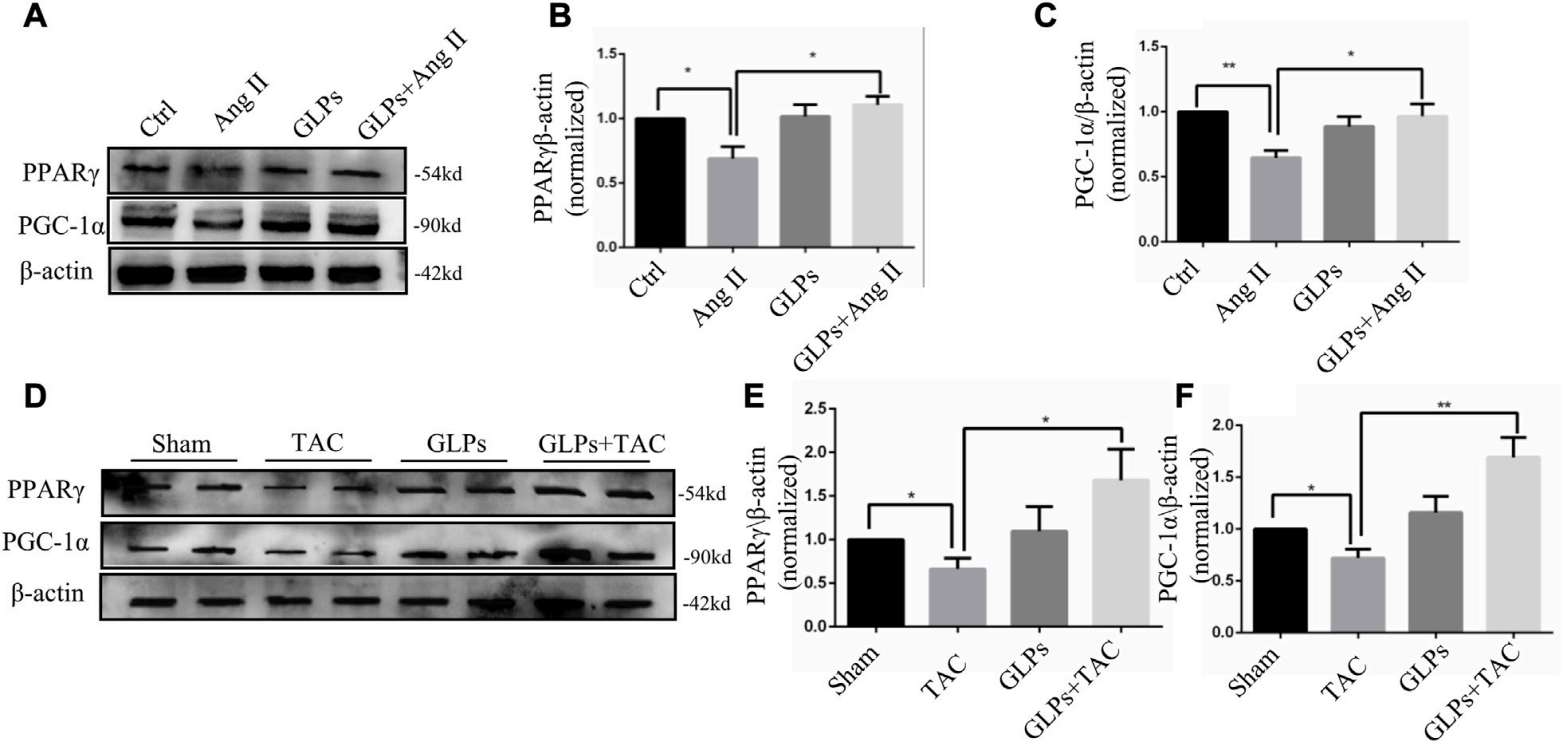
PPARγ and PGC-1α are upregulated by GLPs in both cardiomyocytes and mouse hearts. **(A)** Representative Western blot results for PPARγ and PGC-1α protein levels in extracts from H9C2 cells treated with Ang II (1 µM) and GLPs (50 mg/mL). **(B)** Quantification of the PPARγ protein levels shown in **(A)** (*n* = 3). **(C)** Quantification of the PGC-1α protein levels shown in **(A)** (*n* = 3). **(D)** Representative Western blot results for PPARγ and PGC-1α protein levels in WT and GLP mouse hearts after sham operation or TAC surgery. **(E)** Quantification of the PPARγ protein levels shown in **(D)** (n = 4). **(F)** Quantification of the PGC-1α protein levels shown in **(D)** (*n* = 4). All values represent the means ± SEM, *p < 0.05, **p < 0.01. TAC: transverse aortic constriction; Ang II: angiotensin II; PPAR γ: peroxisome proliferator-activated receptor gamma; PGC-1α: peroxisome proliferator-activated receptor gamma coactivator-1α.; GLPs: *Ganoderma lucidum* polysaccharides.

Studies have shown that cardiometabolic substrates are altered during cardiovascular disease (Wang et al., 2015; Liu et al., 2019). PPARγ and PGC-1α play important roles in stabilizing cardiac metabolism. Therefore, we assessed whether GLPs have an effect on PPARγ and PGC-1α signaling. The results showed that the protein expression levels of PPARγ and PGC-1α were decreased in the Ang II-stimulated group. In contrast, GLP treatment increased PPARγ and PGC-1α expression in the GLP-stimulated group (Figures 4A–C). Next, we examined PPARγ and PGC-1α expression in different mouse heart tissue samples. As shown in Figures 4D–F, PPARγ and PGC-1α levels were decreased in TAC mouse hearts, while PPARγ and PGC-1α levels were increased in GLP-stimulated mouse hearts. These results indicate that GLPs upregulated PPARγ and PGC-1α in both cardiomyocytes and mouse hearts.

### 3.4 GLPs alleviated cardiomyocyte hypertrophy through PPARγ activation

**FIGURE 5:**
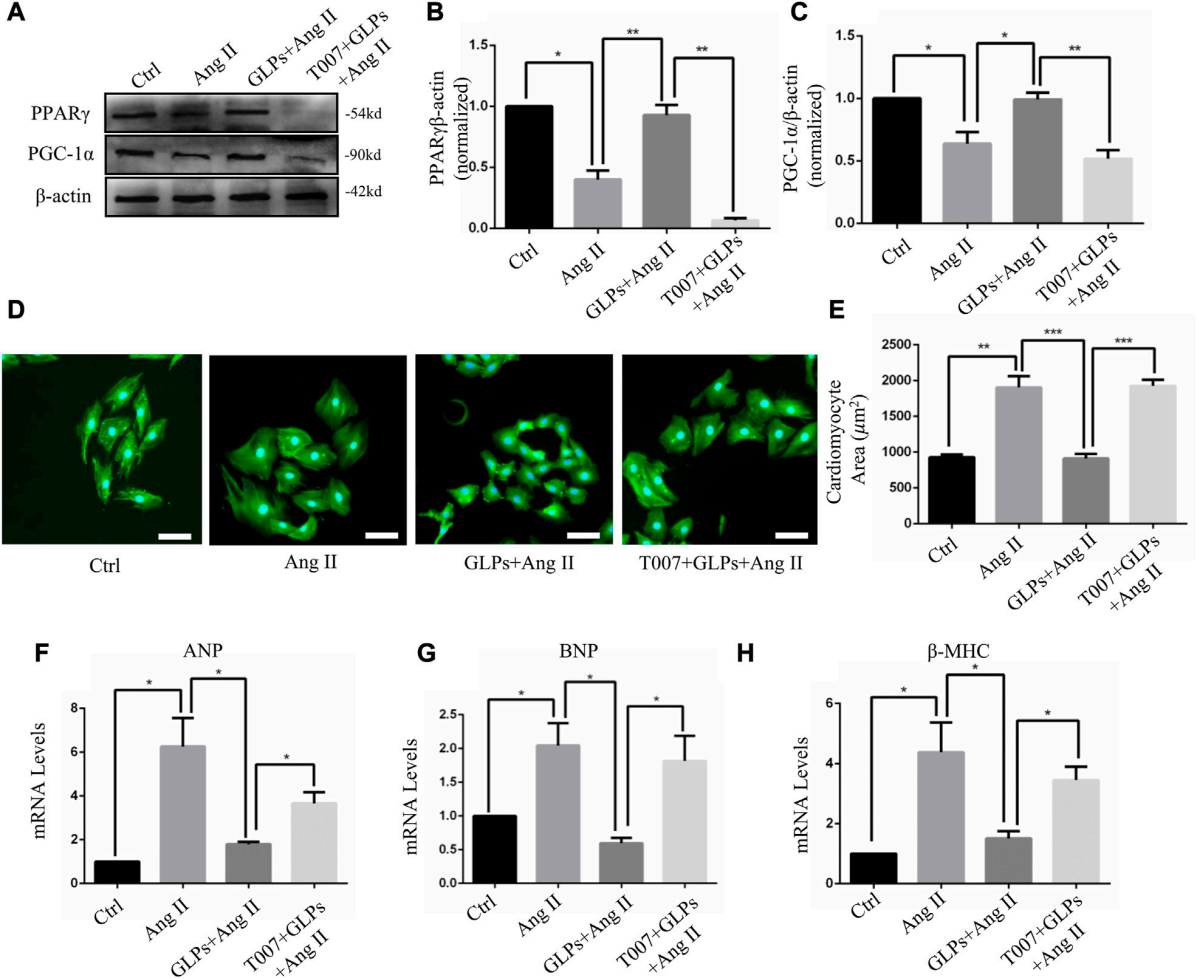
GLPs alleviate Ang II-induced cardiomyocyte hypertrophy *via* PPARγ. **(A)** Representative Western blot results for PPARγ and PGC-1α protein levels in extracts from H9C2 cell treated with Ang II (1 µM) and GLPs (50 mg/mL) and pre-treatment with T0070907 (1 μM-4 h). **(B)** Quantification of the PPARγ protein levels shown in **(B)** (*n* = 3). **(C)** Quantification of the PGC-1α protein levels shown in **(A)** (*n* = 3). **(D)** Representative images of cardiomyocytes treated with Ang II and GLPs and pre-treated with T0070907 (green: α-actinin; blue: DAPI). Original magnification, ×200, scale bars, 50 μM. **(E)** Quantification of cell surface areas (*n* = 50 + cells in each group). **(F)** Real-time PCR analysis of ANP gene expression in H9C2 cells treated with Ang II and GLPs and pre-treated with T0070907. **(G)** Real-time PCR analysis of BNP gene expression in H9C2 cells treated with Ang II and GLPs and pre-treated with T0070907 (*n* = 3). **(H)** Real-time PCR analysis of β-MHC gene expression in H9C2 cells treated with Ang II and GLPs and pre-treated with T0070907 (*n* = 3). All values represent the means ± SEM, *p < 0.05, **p < 0.01, ***p < 0.001. Ang II: angiotensin II; PPAR γ: peroxisome proliferator-activated receptor gamma; PGC-1α: peroxisome proliferator-activated receptor gamma coactivator-1α.; GLPs: *Ganoderma lucidum* polysaccharides; T007: T0070907.

In this section, we explored whether GLP-regulated PPARγ activation is involved in the protective effects of GLPs against Ang II-stimulated cardiomyocyte hypertrophy. After using the PPAR γ inhibitor T0070907 (1 μM), PPAR γ protein expression was decreased (Figures 5A–C). As indicated by α-actinin immunostaining in cardiomyocytes, T0070907 (1 μM) reversed the protective effects of GLPs on cardiomyocyte hypertrophy induced by Ang II (Figures 5D,E). qRT-PCR results indicated that the decrease in hypertrophic gene mRNA levels induced by GLPs was abolished by treatment with T0070907 (Figures 5F–H). Altogether, these results indicate that GLPs alleviated cardiomyocyte hypertrophy through activating PPARγ.

### 3.5 GLPs inhibited pressure overload-induced cardiac hypertrophy through PPARγ activation

**FIGURE 6:**
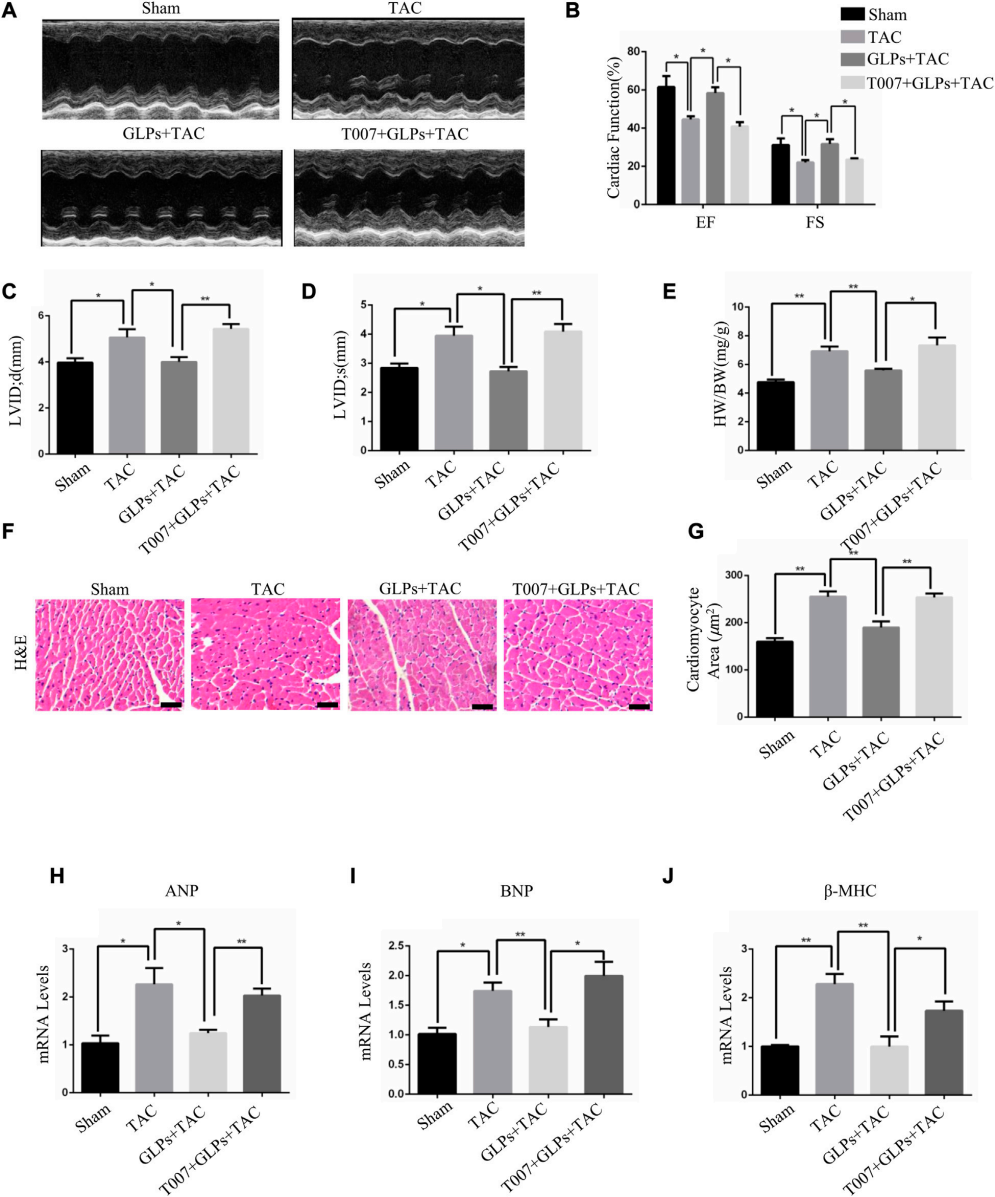
GLPs increase cardiac function and ameliorate cardiac hypertrophy *via* PPARγ. **(A)** Representative M-mode echocardiographic tracings of the left ventricle in mice after sham operation or TAC surgery. **(B)** Quantification of the recovery of EF% and FS% in **(A)** (*n* = 4 per group). **(C)** Quantification of the LVID; d in **(A)**. **(D)** Quantification of the LVID; s in **(A)**. **(E)** Changes in the HW/BW ratio for WT and GLP mice after sham operation or TAC surgery (*n* = 4 per group). **(F)** Histological sections of hearts from WT and GLP mice after sham operation or TAC surgery were stained with H&E to analyze cardiomyocyte size. Original magnification, ×400, scale bars, 100 μM. **(G)** Quantification of cell surface areas (*n* = 50 + cells in each group). **(H–J)** Real-time PCR analysis of hypertrophic markers (ANP, BNP, and β-MHC) in WT and GLP mouse hearts after sham operation or TAC surgery (*n* = 4 per group). All values represent the means ± SEM, *p < 0.05, **p < 0.01. TAC: transverse aortic constriction; EF: ejection fraction; FS: fractional shortening; LVID; d: end-diastolic left ventricular internal dimension; LVID; s: end-systolic left ventricular internal dimension; HW/BW: heart weight/body weight; H&E: hematoxylin and eosin; T007: T0070907; GLPs: *Ganoderma lucidum* polysaccharides.

**FIGURE 7:**
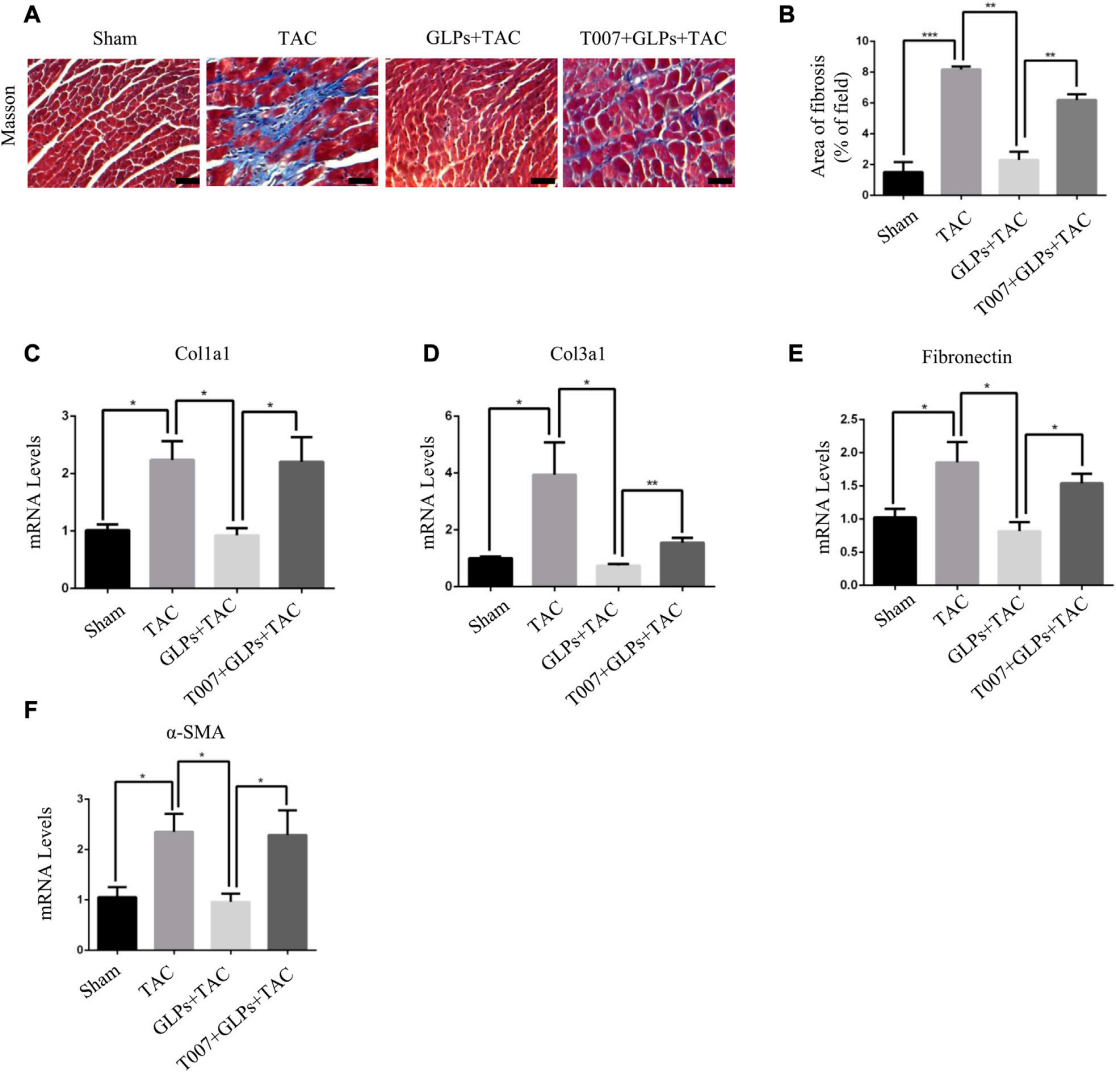
GLPs attenuate cardiac fibrosis induced by TAC *via* PPARγ *in vivo*. **(A)** Histological sections of hearts from WT and GLP mice after sham operation or TAC surgery were stained with Masson’s trichrome to analyze collagen deposition. Original magnification, ×400, scale bars, 100 μM. **(B)** Statistical results for cardiac interstitial fibrosis (*n* = 4 per group). **(C–F)** Real-time PCR analysis of fibrotic markers (Col1a1, Col3a1, fibronectin, and α-SMA) in WT and GLP mouse hearts after sham operation or TAC surgery (*n* = 4 per group). All values represent the means ± SEM, *p < 0.05, **p < 0.01, ***p < 0.001. WT: wild-type; Col1a1: collagen I; Col3a1: collagen III; α-SMA: α-smooth muscle actin; T007: T0070907; GLPs: *Ganoderma lucidum* polysaccharides.

To explore the important role of PPARγ in the reversal of cardiac hypertrophy by GLPs, T0070907 (1 mg/kg/d) was administered to mice. As demonstrated by the echocardiographs shown in Figures 6A–D, GLP treatment inhibited cardiac hypertrophy and failure, evidenced by decreased LVID; s and LVID; d and increased EF% and FS%. However, T0070907 reversed the protective effects of GLPs on TAC-induced cardiac hypertrophy and failure, indicated by increased LVID; s and LVID; d and decreased EF% and FS% (Figures 6A–D). An increased hypertrophic response and increased HW/BW ratios revealed that T0070907 abolished the protective effects of GLPs (Figure 6E). In addition, T0070907 inhibited the reversion effects of GLPs, evidenced by enlarged cell areas and increased expression of hypertrophic genes (ANP, BNP, and β-MHC) (Figures 6F–J). Masson’s trichrome staining revealed that the antifibrotic effect of GLPs was blunted by the PPARγ inhibitor T0070907, evidenced by decreased interstitial fibrosis in T0070907-treated mice (Figures 7A,B). To further determine the antifibrotic effect of GLPs, we measured the mRNA levels of fibrosis-related genes (Col3a1, Col1a1, α-SMA, and fibronectin). T0070907 reversed the GLP-induced downregulation of fibrosis markers in TAC mice (Figures 7C–F). These results show that PPARγ is indispensable for the inhibitory effects of GLPs on cardiac hypertrophy, fibrosis, and failure induced by sustained pressure overload.

## 4 Discussion

For many cardiovascular diseases, cardiac hypertrophy is one of the most important potential pathogenic factors. However, pharmacological treatments to reverse the maladaptive processes and restore cardiac function are limited. Seeking new therapeutic methods and strategies has broad therapeutic prospects. *Ganoderma lucidum* (*G. lucidum*), a rare traditional Chinese medicine, has been widely used in clinical treatment. *Ganoderma lucidum* polysaccharides (GLPs), the active ingredient of *G. lucidum*, have many positive health effects, including anti-tumor, blood pressure lowering, immunity regulation, and liver protection (Jiang et al., 2017; Li et al., 2018; Huang et al., 2019). In our study, we demonstrated that GLPs reversed pressure overload-induced cardiac hypertrophy. First, GLPs inhibited TAC-induced cardiac hypertrophy and fibrosis and rescued cardiac function. Second, GLPs upregulated PPARγ and PGC-1α expression *in vitro* and *in vivo*. Further experiments showed that PPARγ inhibitors blocked the cardioprotective effects of GLPs *in vitro* and *in vivo*.

Increased cardiomyocyte area, thickened ventricular wall, and altered hypertrophic gene expression are the main features of cardiac hypertrophy. Normal cardiac energy metabolism is essential for the maintenance of normal cardiac function, but metabolic disorders often occur in cardiomyocytes during compensatory cardiac growth. PGC-1α and PPARγ play important roles in controlling heart energy metabolism (Di et al., 2018). PPARγ belongs to the peroxisome proliferator-activated receptor (PPAR) nuclear receptor family and is highly expressed in adipocytes, cardiomyocytes, and macrophages. Relevant studies have confirmed that PPARγ activation improved myocardial cell injury caused by ischemia/reperfusion (Zhou et al., 2017), reduced myocardial fibrosis (Ma et al., 2017), reduced cardiomyocyte apoptosis, and inhibited cardiac hypertrophy (Peng et al., 2017). Moreover, regulating PPARγ activation has important applications in clinical treatment. PPARγ activators have been used as hypoglycemic drugs in clinical practice (Wang et al., 2017). Our current study found that PPARγ expression was downregulated during Ang II stimulation in cardiomyocytes and TAC mouse hearts and upregulated during GLP treatment. Importantly, our experiments demonstrated that PPARγ inhibitors reversed the protective effects of GLPs in cardiomyocytes and *in vivo*. Thus, GLPs protect against pressure overload-induced cardiac hypertrophy through PPARγ activation.

Myocardial fibrosis often occurs during the pathogenesis of cardiac hypertrophy and cardiac remodeling (Kong et al., 2014; Segura et al., 2014). Decreasing fibroblast aggregation and activation during cardiac hypertrophy is crucial to slow fibrosis and improve cardiac function (Hou et al., 2013; Yang et al., 2018). In our study, GLPs significantly inhibited myocardial fibrosis, which manifested as reduced fibrosis areas and decreased expression levels of collagen markers. Moreover, a PPARγ inhibitor attenuated the anti-fibrotic effects of GLPs in TAC mice. These results suggest the potential therapeutic effects of GLPS on myocardial fibrosis.

This study has some limitations. The specific mechanism through which GLPs activate PPARγ and the relationship between PPARγ and PGC-1α remain unclear. Additionally, the molecular mechanism of how GLPs regulate cardiac hypertrophy is not well understood. Further studies are needed to elucidate the detailed mechanisms by which GLPs prevent pathological cardiac hypertrophy.

## 5 Conclusion

Overall, our study demonstrates that GLPs can protect against pressure overload-induced cardiac hypertrophy through PPARγ activation. The PPARγ inhibitor T0070907 reversed the protective effects of GLPs on cardiomyocyte hypertrophy. Furthermore, *in vivo*, T0070907 blocked the preventive effects of GLPs on cardiac hypertrophy and heart fibrosis. This study provides important theoretical support for the potential of GLPs in the treatment of pathological cardiac hypertrophy and expanding the use of GLP in the treatment of pathological cardiac hypertrophy and heart failure.

## Data Availability

The original contributions presented in the study are included in the article/supplementary material, further inquiries can be directed to the corresponding authors.
